# Biotic interactions explain seasonal dynamics of the alpine soil microbiome

**DOI:** 10.1093/ismeco/ycae028

**Published:** 2024-02-28

**Authors:** Anna Maria Fiore-Donno, Jule Freudenthal, Mathilde Borg Dahl, Christian Rixen, Tim Urich, Michael Bonkowski

**Affiliations:** Institute of Zoology, University of Cologne, Zuelpicher Str. 47b, 50674 Cologne, Germany; Institute of Zoology, University of Cologne, Zuelpicher Str. 47b, 50674 Cologne, Germany; Institute of Microbiology, University of Greifswald, 17489 Greifswald, Germany; WSL Institute for Snow and Avalanche Research SLF, 7260 Davos Dorf, Switzerland; Climate Change, Extremes and Natural Hazards in Alpine Regions Research Centre CERC, 7260 Davos Dorf, Switzerland; Institute of Microbiology, University of Greifswald, 17489 Greifswald, Germany; Institute of Zoology, University of Cologne, Zuelpicher Str. 47b, 50674 Cologne, Germany

**Keywords:** biotic interactions, alpine ecology, protists, metatranscriptomics, soil food web, soil ecology, community ecology

## Abstract

While it is acknowledged that alpine soil bacterial communities are primarily driven by season and elevation, there is no consensus on the factors influencing fungi and protists. Here we used a holistic approach of the microbiome to investigate the seasonal dynamics in alpine grasslands, focusing on soil food web interactions. We collected 158 soil samples along elevation transects from three mountains in the Alps, in spring during snowmelt and in the following summer. Using metatranscriptomics, we simultaneously assessed prokaryotic and eukaryotic communities, further classified into trophic guilds. Our findings reveal that the consumers’ pressure increases from spring to summer, leading to more diverse and evenly distributed prey communities. Consequently, consumers effectively maintain the diverse soil bacterial and fungal communities essential for ecosystem functioning. Our research highlights the significance of biotic interactions in understanding the distribution and dynamics of alpine microbial communities.

## Graphical Abstract

**Graphical Abstract:**
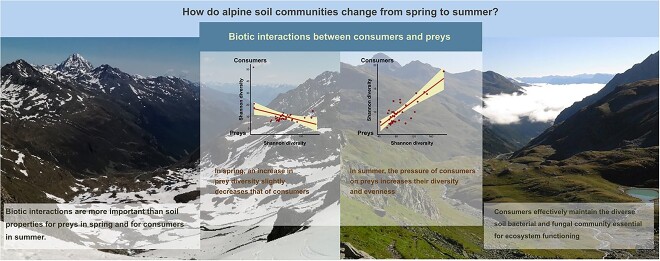

We investigated the seasonal dynamics and interactions of soil food webs in the Alps. Our metatranscriptomic results from 158 samples reveal (i) that biotic interactions can explain more variation in the microbial communities than topographic and edaphic variables; (ii) a seasonal dynamic in biotic interactions: consumers pressure on preys increases from spring to summer; (iii) that, contrary to current models, there is no shift in communities during thaw.

## Introduction

Understanding the seasonal dynamics of the soil microbiome is an important step towards modelling the effects of climate warming, i.e. whether and how the balance between the carbon stored in the soil and the CO_2_ released to the atmosphere will be altered [1]. It is not yet clear how the main components of the soil food web—bacterial and fungal primary decomposers and their main consumers (predatory bacteria, heterotrophic protists and bacterivorous and fungivorous nematodes) —are structured in alpine regions, and in particular which of the abiotic drivers, elevation or season, has a preponderant influence [2, 3]. Along altitudinal gradients, bacterial and fungal diversity generally decreases with elevation [4], while protistan diversity increases [3]. While most studies agree on the importance of pH and elevation in shaping bacterial communities in general, the unexplained variance is usually greater for fungi and protists [3-5].

Recently, models that include biotic interactions in addition to abiotic environmental variables have been shown to explain significantly more variation in microbial metacommunity assembly [6-8]. Biotic interactions, such as competition and predation, can promote the coexistence or exclusion of species [9]. Thus, the inclusion of biotic interactions is of great importance for process-based understanding and prediction of ecological responses [10]. For instance, ecosystem services, such as C and N cycling, in which soil microbes play an important role, can be altered by consumer–prey interactions [11]: It has been suggested that in soil microbiomes with higher productivity there is increased predation on lower trophic levels, influencing carbon flow through the belowground food web [12]. In addition, predation contributes to the formation of the soil microbial necromass, the importance of which, accounting for up to 50% of soil organic matter, has only recently been recognized [13]. It is therefore crucial to emphasize the role of dynamic trophic relationships, also as a cause of microbial mortality [14].

**Figure 1:**
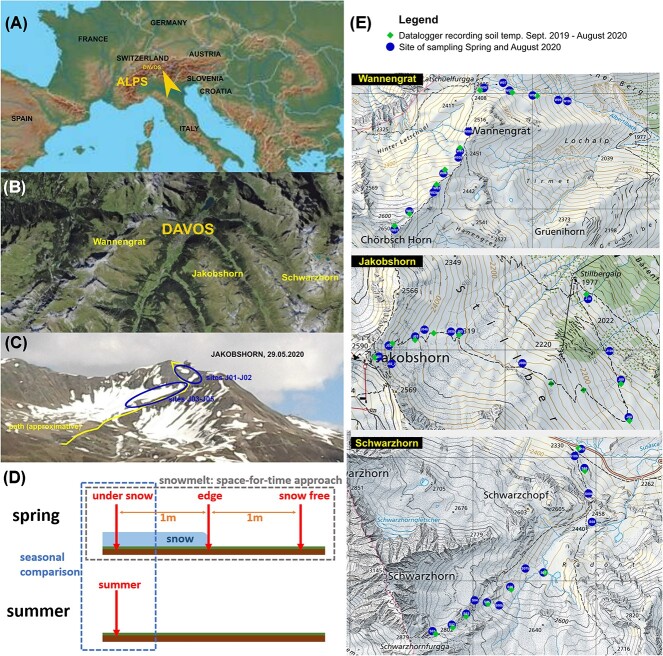
Maps of the collection sites and sampling scheme. (A) The Alps and Davos in Europe. (B) The three mountains where the study took place. (C) View of the sites at the top of the Jakobshorn during the spring sampling. (D) A scheme of the sampling design per site. (E) Sites and dataloggers position along the altitudinal gradient in each mountain.

Our study aimed at disentangling the interactions of seasonal, topographic, edaphic and biotic factors and how they shape the soil microbiome. To this end, we used metatranscriptomics to simultaneously assess the prokaryotic and eukaryotic microbial diversity in alpine grasslands in Switzerland, near Davos (Fig. 1A and B), at a regional scale (158 samples on three different mountains), taking into account elevation (altitudinal transects from c. 1900 to 2800 m a.s.l) and season (spring and summer; Fig. 1C-E). Small subunit ribosomal RNAs (16S and 18S) were used to identify the bacterial, archaeal, fungal, metazoan, and protistan communities to the genus level. To investigate biotic interactions, identified genera were further classified, where possible, according to nutrition and lifestyle.

We addressed the following key questions: (i) which are the dominant components of the soil microbiome during snowmelt and what is the fate of these communities in summer? (ii) Do consumers contribute to the seasonal turnover of the bacterial and fungal communities? (iii) Which are the main drivers of alpine soil communities, topography, soil properties, seasonality or biotic interactions? We hypothesized that consumers would play a major role in shaping prey communities, with an increase in consumers and a decrease in preys during the season, with season playing a more important role than elevation. Finally, we hypothesized that each trophic guild would differ in its response to environmental and seasonal changes.

## Materials and methods

### Sampling design

The study area is located around the town of Davos in the eastern central Alps in Switzerland (Fig. 1). The climate is characterized by an average annual temperature of 3.5 °C, 193 frost days, a total annual precipitation of 1022 mm and fresh snow on 69.1 days per year [15]. Sampling was designed to capture the biogeochemical changes that occur during snowmelt in alpine grasslands. For this, we selected 15–16 sites per mountain in spring 2020, where snow patches were still present. At each site, we collected three soil samples constituting a time series: the first sample under the snow, the second at the edge of the patch, i.e. exactly during snowmelt, and the third in soil recently cleared of snow (Fig. 1D). To assess a medium-scale reproducibility of our data, we repeated the same scheme in three mountains (Fig. 1E), while the altitudinal gradient from 1972 to 2816 m would allow comparing the biogeochemical data between early and later snowmelt periods. To establish a seasonal cycle, we sampled again in August 2020 the same sites that were under snow in spring.

### One year of soil temperature records

In each mountain, 10 dataloggers (ibutton DS1922L, Maxim Integrated Products, San Jose, CA, US) set up to record the temperature every 7200 s to an accuracy of 0.0625 °C were placed in early September 2019 c. every 50 m along the elevation gradient, and recovered in August 2020, as previously described (Rindt et al. 2023) (Fig. S1). Coordinates and heights were determined using a GPS (Trimble Geo XH 6000, Trimble Inc. Sunnyvale, CA, US) with an accuracy of a few cm (Table S1).

### Soil sampling

Spring sampling took place between 19.5 and 24.6.2020; the three highest sites on the Schwarzhorn could only be sampled on 6 July. The summer sampling took place from 20 to 25 August. Soil temperature at the time of collection was recorded using the described dataloggers, inserted at a depth of c. 5–8 cm, and left for c. 10 min. to record the temperature to an accuracy of 0.0625 °C (Table S1). Two to 4 g of wet soil for RNA extraction were collected with a clean plastic spoon and immediately placed in a sterile, RNAse-free 15 ml plastic tube containing 6.5 ml of Life Guard soil RNA (Qiagen GmbH, Hilden, Germany) and stored in an insulated box with cooling packs. The samples were stored at −20 °C as soon as we returned to the WSL Institute in Davos. They were not allowed to thaw until extraction. Soil (c. 200 g) for determining edaphic properties was collected with a clean metal spoon and stored at 4 °C in a polyethylene bag.

### RNA extraction, reverse-transcription, library preparation and sequencing

Prior starting the following steps, great care was taken to work in an RNAse-free environment, notably by treating all objects that would come into contact with the samples with RNaseZap, an RNase decontamination solution (Sigma-Aldrich, MO, USA). The tubes containing the soil samples were thawed, centrifuged, and the buffer removed. Circa 1 g of wet soil was removed with a spatula and transferred to the RNeasy PowerSoil Total RNA kit vials (Qiagen GmbH, Hilden, Germany). The manufacturer’s protocol was strictly followed, except for the disruption step, which was carried on an MP Biomedicals FastPrep-24 homogenizer for 30 s at 5 m/sec. The RNA was eluted in 50 μl of SR7 buffer, with the addition of 1 μl of recombinant RNasin ribonuclease inhibitor (Promega, Madison, WI, USA). DNAs were digested with DNAse I (New England BioLabs, MA, USA) and proteins and small RNAs were removed using the Megaclear kit (Invitrogen, CA, USA), following the manufacturer’s protocol. Samples were eluted with 50 μl of preheated elution buffer, and quantified using a Qubit 30 Fluorometer (Invitrogen, CA, USA) using 2 μl of the RNA in the high sensitivity buffer. Quality was estimated with a 2100 Bioanalyzer (Agilent, CA, USA) using the Prokaryote Total RNA Nano assay. Samples with a concentration < 11 ng/μl were precipitated with 1:10 volume of 5 M ammonium acetate and washed with ethanol, according to the protocol of the Megaclear kit, to reach an RNA concentration > 10 ng/μl. Libraries were prepared using the NEBNext Ultra II Directional RNA Library Prep Kit (New England Biolabs, Ipswich, MS, USA) without rRNA removal or mRNA selection. The incubation time of the first strand cDNA synthesis at 42 °C was increased from 15 to 50 min. To select cDNA fragments of 370–600 bp after the second strand synthesis, the fragmentation time was reduced to 10 min. The library size option “400 bp” was selected and the final libraries were amplified with 12 polymerase chain reaction (PCR) cycles. The libraries were sequenced in a single complete run of NovaSeq SP FC (Illumina Inc., San Diego, CA, US), length of paired sequences of 250 bp, at the Cologne Genomic Centre, University of Cologne, Germany.

### Sequence analyses - filtering and identification

We obtained 1.02 × 10^9^ raw sequences, which were submitted to the PhyloFLASH processing pipeline using default settings [16]. In brief, the pipeline identifies SSU rRNA sequences by aligning unpaired sequences to a filtered SILVA database (v.138, NR99), from which LSU, low-complexity, and cloning vector fragments have been removed. SSU sequences were identified using a short sequence aligner for DNA and RNA-seq data [17], with the default setting of a minimum identity of 70%. Bacteria and Archaea were taxonomically assigned by taking the last common ancestor of the taxonomy strings of all the hits. Eukaryotic forward and reverse SSU sequences were assembled using FLASH [18] and low quality sequences were filtered out with default settings. Eukaryotic sequences were identified to the genus level using a slightly modified PR2 database [19], using Blast + [20] with an e-value of 1e-10 and keeping only the best hit. Unicellular eukaryotes were classified as protists, and additional information on lifestyle (free-living, plant, or animal parasite) and nutrition (heterotroph, autotroph, or mixotroph) was added whenever possible, according mostly to [21]. Nematodes were classified as eukaryvore, bacterivore, plant-feeding, fungivore, animal parasite, or omnivore [22, 23]. To assess the biotic interactions, we considered broadly defined groups of consumers, i.e. bacterial predators—the phyla Myxococcota and Bdellovibrionota (94.7% of the assemblage), heterotrophs and free-living protists (4.3%), and free-living nematodes (1%), and preys, i.e. all other bacteria (99.2%), fungi (0.77%), and autotrophic protists (unicellular algae, 0.03%) (Table S2).

### Edaphic properties and vegetation survey

Soil water content, pH, soil organic C and total N, soil microbial biomass C and N, dissolved organic C, and total dissolved N were measured as previously described [24]. Vegetation was recorded from June to August in 2020 and 2021, within a 40 cm diameter circle around the spot where the soil sample was taken. All vascular plants rooted within the surface were identified according to [25], and the percentage of the surface they covered was estimated (Table S1).

### Statistical analyses

All statistical analyses were carried out within the R environment (R v. 4.1.3) [26] on the taxonomic abundance/sample (Table S2) and on the sample topographic, edaphic, and biotic characteristics (Table S1). Unless otherwise specified, community analyses were performed with the vegan package 2.5–7 [27]. To assess whether more sampling and sequencing effort would have revealed more richness, we performed an analysis based on accumulation curves (function *specaccum*) and rarefaction curves (package and function iNEXT 3.0.0), using the abundance table, with a 97% confidence interval, 50 bootstraps and 50 knots; the latter function also calculates species richness (observed and estimated) and sample coverage. Exponential Shannon indices were calculated with the function *renyi* (with the hill parameter, on sample-standardized data, with function *decostand*, method “total”). Significant differences in sample-standardized sequence counts, alpha diversity and evenness between seasons were determined by analysis of variance and Tukey tests (package agricolae 1.3-5, function *aov* and *HSD.test*) with a *P*≤.05, while correlations between the same data were determined with the function *lm*.

Beta diversity between mountains, altitude and snow coverage in spring was inferred by Principal Coordinate Analysis (function *cmdscale*), using Bray–Curtis dissimilarities (function *vegdist*, method “bray”) on the sample-standardised taxa of interest (Bacteria, Archaea, protists, Fungi and the functional groups consumers and preys), then plotted with the package ggplot 2 3.3.5. Principal component analysis revealed an influence of altitude and mountain on bacterial beta diversity, but this effect was mainly driven by the three highest sites in the Schwarzhorn (Fig. S2). When these outliers were removed, a decrease of the variation explained by the first axis was observed (40.1 to 31.3%), and a trend for altitude was visible. Fungal, metazoan, protistan and consumer communities displayed no clear trend with increasing elevation (Fig. S2). Variation partitioning (function *varpart* applied to the Hellinger-transformed taxa dataset and using RDA, function *rda*) was used to assess the proportion of beta diversity explained by each of the factors mountain, altitude and spring snow coverage.

Differential abundances of the most abundant taxa across seasons were calculated with the package DESeq2 1.30.1 [28]; DESeq objects were created with function *DESeqDataSetFromMatrix* and normalised (function *estimateSizeFactors*), then the differential expression was calculated using *DESeq* with the parameters minReplicatesForReplace = Inf, sfType = “poscounts”. Results with an adjusted p-value <0.01 and an absolute log2fold >0.5 were considered as significant and plotted with ggplot2 (*geom_segment*).

Distance-based redundancy analysis (dbRDA, function *dbrda*) was conducted to describe the influence of environmental factors (scaled with function *scale*) on the distribution of the abovementioned taxa of interest (standardized by samples as above). The most influential variables were identified with the function *ordistep* based on the Akaike Information Criterion, and the resulting model tested with *anova*.

To estimate the proportion of variance accounted for by topographic (mountain and altitude), biotic (Shannon vascular plants, Shannon preys or consumers), and edaphic (soil temperature, water content, pH and organic C) factors in the diversity of consumers and preys by season, variance partitioning analyses were performed. The variance attributed to each category of factors (topographic, biotic, and edaphic) and their intersections was estimated using the R^2^ of the linear models (function *lm*) of log-transformed Shannon indices of preys or consumers versus the other categories of factors.

To investigate associations between consumers and preys and among consumers, network analyses were performed for each season on the most abundant taxa (Table S3), further filtered by prevalence, i.e. selecting taxa that were present in more than one third of the sites. The filtered out taxa were binned into a “pseudo-taxon”, which was used for inferences to avoid altering the ratio between taxa, as recommended by [29], but was not shown in the final results. The co-occurrence network was calculated at the genus level using FlashWeave [30], a package implemented in julia v.1.7.1 [31] with parameters for homogeneous data (sensitive = true and heterogeneous = false). Co-occurrences were estimated in several steps: first, for each genus, all directly associated neighbours were computed; then, individual neighbours were connected through a combinatorial strategy to form a global association graph, with an optimized sequence of statistical tests for conditional independence. The final set of directly associated neighbours contained only genera that were conditionally dependent on each other [30]. The network was then summarized (using R) to higher taxa and functions and visualized using Cytoscape v.3.9.1 [32], which was also used to analyse original networks (not summarized). We excluded self-loops, nodes with only one association and associations within preys.

## Results

### Metatranscriptomics

From the 158 collected soil samples, we obtained more than one billion RNA sequences (on average c. 2.5 million/sample), resulting in c. 8000 identified taxa at the genus level (on average 2349 per sample) (Table S4). Rarefaction curves describing the observed number of genera as a function of the number of sequences (Fig. S3) suggested that our sequencing effort was sufficient, confirmed by the estimated sample coverage (between 99.95 and 100%, Table S2). Therefore, and because all samples were sequenced in a single run, we considered that differences in the number of sequences between, e.g. taxonomic or trophic groups, was informative per se. Accumulation curves describing the number of genera as a function of the number of samples, binned by altitude, mountain, and snow coverage (Fig. S4) did not reach a plateau and showed slight differences between environments, thus indicating differences in beta diversity.

### Taxonomic and functional diversity

**Figure 2:**
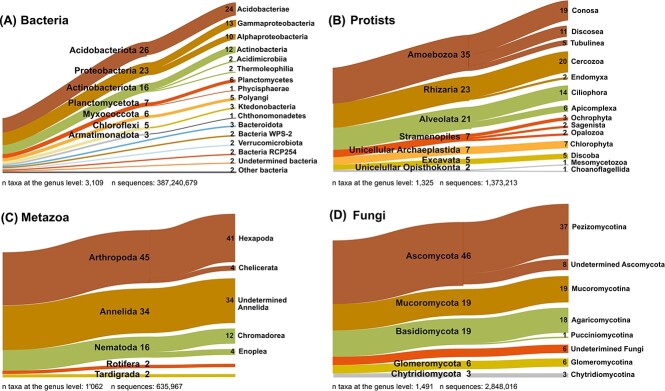
Sankey diagrams showing the relative proportion of high-rank taxonomic groups, based on percentage of sequences identified to the genus level. Taxa <1% are not shown. (A) Bacteria. (B) Protists. (C) Metazoa. (D) Fungi.

Prokaryotes dominated the total assemblage with 98.02% of the identified taxa, of which only 0.06% were Archaea (Table S2). Among bacteria, it is noteworthy that the predatory Myxococcota represented almost 6% of the bacterial genera (Fig. 2a and Note S1), making it the main consumer in soil, surpassing the heterotrophic protists in abundance. Among eukaryotes, fungi and metazoa accounted each for 36% of the SSU rRNAs, protists for 17% and multicellular plants for 11% (Table S2). More than a third of the protistan sequences were assigned to the phylum Amoebozoa (35%; Fig. 2B), including the very elusive slime mould Myxogastria. The phylum Rhizaria (23%) was in majority composed of Cercozoa (20%). Our functional classification by nutrition showed that 88% of the protists were heterotrophs, 8% autotrophs, and 2% mixotrophs. In terms of lifestyle, 90% were free-living, 7% were animal parasites, and 2% were plant parasites (Table S2).

Among the animals (Metazoa), insects (41%), and ringed worms (34%) were most abundant (Fig. 2C), followed by nematodes (16%). Nematodes were classified as bacterivores (29%), plant-feeding (25%), fungivores (16%), eukaryvores (10%), omnivores (3%), and animal parasites (1%; Table S2).

The fungi were dominated by Ascomycota (46%; Fig. 2D), mainly Pezizomycotina (37%), which also makes up most of Ascomycota in terms of described species. Of the 53% of the fungi that could be functionally identified, saprotrophs were the largest group (35%), followed by plant parasites (7%), endomycorrhizals (6%), ectomycorrhizals (5%), lichenized (5%), ericoid-mycorrhizals (2%), and other parasites and endosymbionts each representing <1% (Table S2).

### Soil temperatures and edaphic parameters

During our study, the snow cover lasted on average 228 days (max. = 286, min. = 186, sd = 21.2), 22 days longer on the high than on the low altitudes (Table S1). Soil temperatures were on average 2 °C lower on the high than on the low altitudes (Table S1) and showed a day/night variation before and after the snow covered the soil; during winter under the snow cover, temperatures were stable and remained constantly >0 °C (Fig. S1). Temperatures recorded during the sampling increased during snowmelt and further increased in summer, mirrored by an opposite trend in soil water content. The pH did not significantly vary during snowmelt. Microbial biomass and dissolved C increased during and immediately after snowmelt, as well as from spring to summer (Table S1) [24].

### Dynamics during snowmelt and from spring to summer

**Figure 3:**
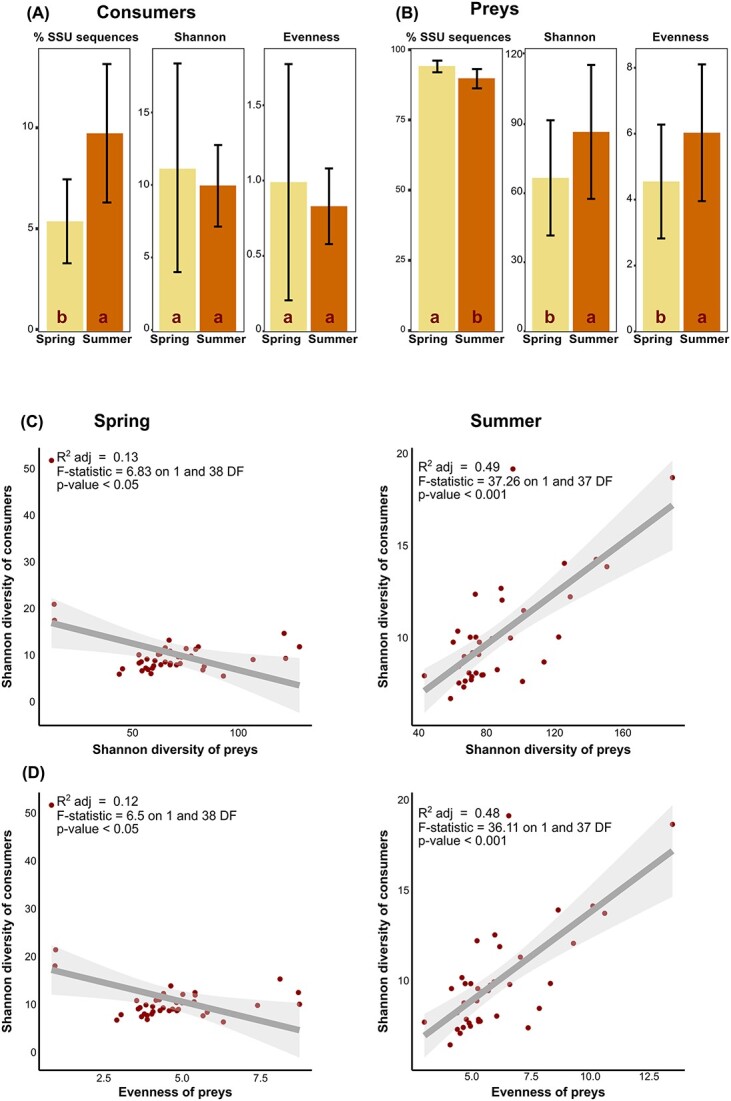
(A, B) seasonal variation in the standardized SSU rRNA sequences, diversity (Shannon index) and evenness, from the sample under the snow in spring to summer. Significant changes (analysis of variance, *P*-value ≤.05) are indicated by “a” (higher) and “b” (lower). Standard errors bars are shown. (A) Consumers (predatory bacteria, heterotrophic and free-living protists, selected nematodes). (B) Preys (non-predatory bacteria, fungi and autotrophic protists). Consumers increase from spring to summer (without significant changes in diversity or evenness); preys decrease, while their diversity and evenness increase. (C, D) linear correlations between consumers and preys (y and x axis, respectively), in spring and summer. (C) Shannon indices. (D) Shannon index of consumers versus evenness of preys. Dots = samples. Grey surface = 95% confidence interval.

In spring, despite sharp environmental changes across the snowmelt gradient, no significant differences in the abundance, diversity or evenness of the communities of consumers and preys were observed (Fig. S5). In contrast, abundance, diversity, and evenness varied between the sample collected under the snow in spring and the sample collected at the same spot in summer, with differences between functional guilds. For instance, the standardized counts of consumer SSU rRNAs sequences was significantly more abundant in summer than in spring, but no significant differences in diversity and evenness were found (Fig. 3A—expressed as percentage of total sequences). In contrast, the prey SSU rRNAs were more abundant in spring, but preys were more diverse and even in summer (Fig. 3B). Changes in diversity and evenness of consumers and their potential prey were correlated. There were negative correlations between the diversity and the evenness of consumers and those of preys in spring (Fig. 3C and D). On the contrary, the same negative correlations were strong and positive in summer (Fig. 3C and D). We questioned whether the increase of consumers in spring was related to the main abiotic changes occurring from spring to summer, i.e. a warmer and drier soil; we found positive linear correlations between soil temperature and water content and the richness of Myxococcota (F_[1, 156]_ = 7.6, *P* = .007) and Rhizaria (F_[1, 156]_ = 14.27, *P* < .001), but not for that of Amoebozoa.

### Most influential environmental parameters

We tested how consumers and preys were responding to different factors, categorized as topographic (altitude, mountain), edaphic (water content, pH, soil temperature, and organic C), and biotic (coverage and diversity of the vascular plants, diversity of bacteria, fungi, protists, and consumers). Models (dbRDA) were estimated during spring snowmelt and from spring to summer (Table S5). The models retained pH and prey diversity as the main drivers for consumers in summer; remarkably, the prey diversity was more important than pH in summer (Table S5). Preys were also more influenced by the diversity of consumers under the snow than by topographic and edaphic factors. Bacteria were influenced by altitude and edaphic parameters in different combinations, depending on the sample; biotic factors had little influence. Less variation was explained in models estimated for protists, with low F values and inconsistent results between samples. Fungi did not respond to topographic nor edaphic factors, but sporadically to bacterial and protistan diversity. The mountains had no effect in this analysis (Table S5).

For a reliable interpretation of our results, it was important to test the influence of our sampling design, i.e. the four samples per site (Fig. 1D), the altitudinal gradient (Fig. 1E), and the three mountains (Fig. 1B) on the microbial communities. The first axis of principal component analysis only explained 31.3% of the variation of bacteria, with a slight effect of altitude. As in the dbRDA models, the three mountains had no influence, and the fungal, protistan, and consumer communities displayed no clear clustering trend with respect to the parameters tested (Fig. S2).

### Biotic interactions

**Figure 4:**
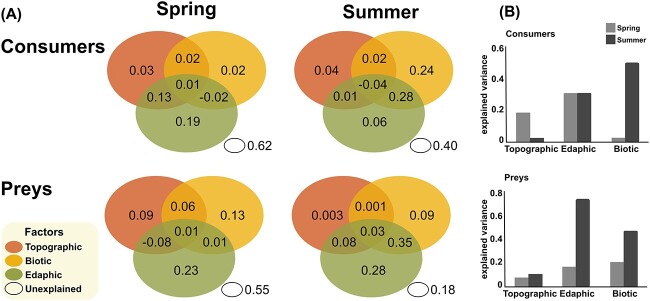
Relative influence of topographic, biotic and edaphic factors on the diversity of consumers and preys, by season. (A) The proportion of variance explained by topographic (upper left ellipse), biotic (upper right) and edaphic (bottom) factors, and the unexplained variance. (B) Comparison of explained total variance by group of factors (the sum of each ellipse), in spring and summer.

Variation partitioning, conducted to disentangle the relative influence of topographic, edaphic, and biotic factors, indicated a clear seasonal trend—all factors together explained more variance in summer than in spring, for both consumers and preys (Fig. 4A). A seasonal increase in the relative influence of biotic factors and biotic + edaphic factors on community variance was observed, higher for consumers (c. 17 times) than for preys (c. 2 times; Fig. 4B).

**Figure 5:**
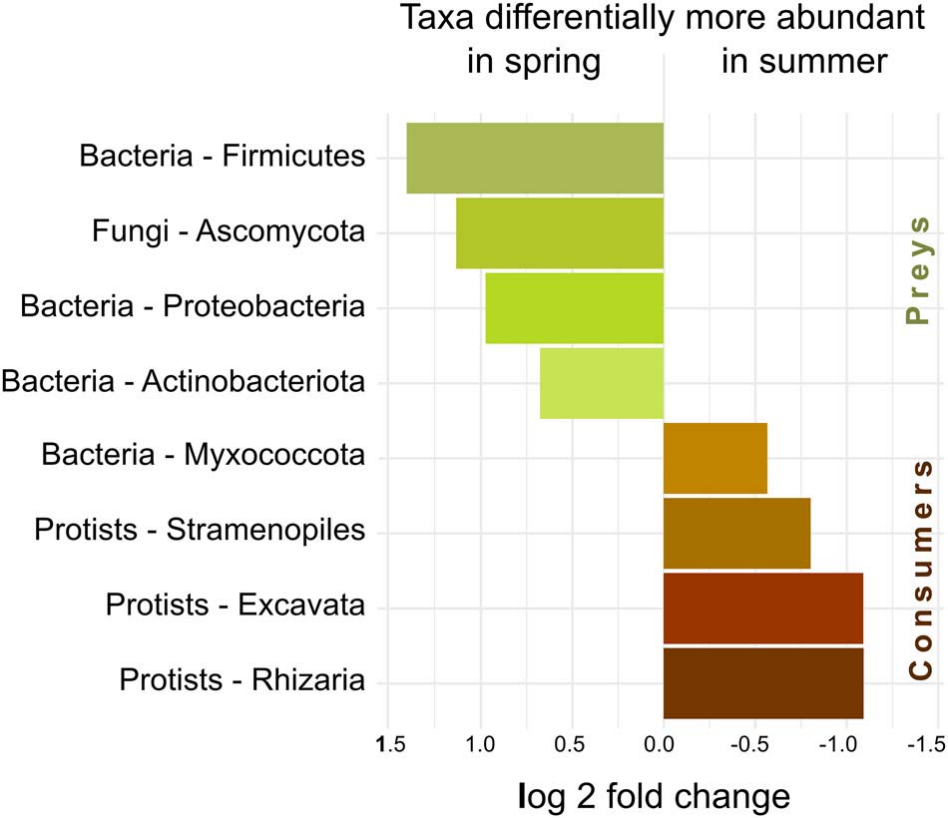
Differential abundances. Taxa were filtered according to abundance and presence in one-third of the samples. All taxa differentially more abundant in spring and summer are preys and consumers, respectively.

Differential expression analysis revealed which taxa were significantly more abundant in spring or in summer. Strikingly, all groups selected by this analysis as more abundant in spring were preys (Bacteria: Firmicutes, Actinobacteria and Proteobacteria, Fungi: Ascomycota), whereas those more abundant in summer were consumers (Bacteria: Myxococcota, protists: Rhizaria, Stramenopiles, and Excavata; Fig. 5).

**Figure 6:**
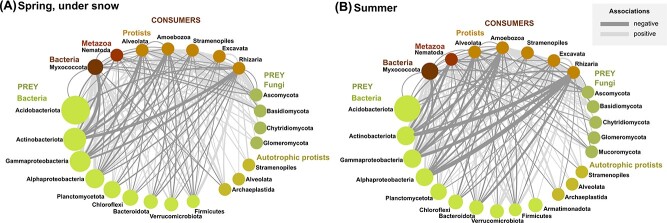
Co-occurrence networks of abundant phyla of consumers and preys. (A) Spring, under the snow. (B) Summer. The size of the nodes (dots) is proportional to the number of sequences. Edges (connecting lines) represent positive (light grey) or negative (dark grey) correlations, with line width proportional to the number of correlations. Self-loops, taxa with a single edge and connections between preys are not shown.

The co-occurrence network confirmed the dominant role of biotic interactions between consumers and preys in summer, showing more associations—and more negative ones—in summer than in spring (Fig. 6A and B, Table S6). In summer, Rhizaria stood out among the consumers with strong negative associations with several major bacterial phyla, i.e. Actinobacteria and Alpha- and Gammaproteobacteria. No seasonal differences were found in the networks of associations between preys, which reflect competitive or facilitative interactions or the sharing of ecological niches (Fig. S6). However, there were no noticeable differences in the structure of the networks, which were only affected by shifts in the relative proportions of the associations.

## Discussion

### How biotic interactions shape the soil communities

The opposite and correlated changes in SSU rRNA relative abundance, diversity and evenness of consumers and preys from spring to summer strongly suggest an effect of predation: the increase of grazers’ abundance reduces the prevalence of preys, whilst increasing their diversity and evenness—predators shape the preys’ communities (Figs 3, 5 & 6). It has been repeatedly demonstrated, particularly in aquatic environments, that predation prevents competitive exclusion—i.e. the dominance of few better adapted species profiting from the resources of a given habitat [33-35]. Thus, as observed here, the increase in consumers’ abundance leads to an increase in preys’ evenness and diversity, while without predators, competition for resources may result in the domination of fewer species [36]. The significance for ecosystem functioning is unmistakable: highly uneven communities, with an extreme dominance by few species, are less resistant to environmental stress [37]. Thus, both the number and relative abundances of species must be sustained to achieve a vigorous ecosystem functioning [38]. In this study, we show that in alpine grasslands with a constant snow cover in winter, the increase in consumers from spring to summer effectively contributes in maintaining a diverse bacterial and fungal community.

The communities of consumers and preys were organized in highly interconnected networks, with stronger negative interactions occurring in summer (Fig. 6). This, in addition to the previous results, likely reflects predator–prey interactions. Among protistan consumers, Cercozoa and Amoebozoa display the strongest negative associations with the major bacterial phyla. Supporting a consumer effect—but not proving it—the dominant cercozoan taxon is bacterivorous (Glissomonadida, 43% of all cercozoan sequences; Table S2). Negative associations between Cercozoa and Actinobacteria (important polysaccharide decomposers) [39] and Alphaproteobacteria were also observed in the rhizosphere [40, 41] where the glissomonads were most abundant (compared to bulk soil and litter) [42]. A link between the decrease of Actinobacteria and the grazing of heterotrophic protists was also suggested [43]. However, negative associations may be solely abiotic, e.g. due to opposite sensitivity to environmental conditions, or biotic but driven by prey defences, such as Actinobacteria secreting secondary metabolites to evade predation [44]. In addition to predation, competitive interactions have been shown to be a major driver in bacterial community composition [45]. For instance, fungal–bacterial competition explained 32% of the variance within planktonic bacterial communities [6]. Accordingly, we observed intricate networks between preys, not or only slightly affected by season (Fig. S6). As our aim was to observe if and how biotic interactions changed between spring and summer, we performed our analyses using a software [30] that attempts to remove associations driven by environmental data, e.g. shared niches. Accordingly, the networks calculated with (Note S1) and without (Fig. 6) environmental data were quite similar.

Our findings are in line with multiple studies which demonstrate that bacterial communities are consistently driven by edaphic or topographic parameters (pH, altitude, water content, and organic carbon), whereas eukaryotic communities display weaker trends in response to environmental gradients in comparison to bacteria (Fig. S2 and Table S5) [4, 46]. Subsequently, while it is generally agreed that bacterial communities are predominantly driven by pH, there is no consensus as to a single main driver for fungal and protistan communities [5, 46, 47]. Indeed, distinct taxa or functional groups of fungi and protists have different optima along environmental gradients and therefore are differentially affected by seasonal and/or altitudinal changes [4, 5, 47, 48]. Similarly, protistan trophic guilds, e.g. consumers, parasites, and phototrophs, differentially respond to altitude and edaphic factors [49]; it is noteworthy that only 40% of the species turnover in these communities could be explained by abiotic factors [47].

It logically follows that the drivers of the fungal and protistan biogeographies must be sought elsewhere. Our models indicated that the most influential response was between functional guilds—in particular, consumers displayed a strong and consistent response to prey diversity, and more so in summer (Table S5). Additionally, variation partitioning (Fig. 4) demonstrated that the combined influence of biotic and edaphic factors explained the largest share of variance in consumer and prey communities. Thus, the intricate interplay between the environment and competitive and/or predatory interactions is best observed when functional guilds are taken into account.

### Changes during snowmelt

Our findings reveal that alpine soil microbial communities, including protists, undergo gradual changes from spring to summer, without any sudden shift during snowmelt (Fig. S5), despite the drastic environmental changes occurring during thaw [24]. The soil microbial biomass in alpine grassland typically attains its annual peak during winter, just before snowmelt [50]. In soils frozen during winter, the soil microbial biomass suddenly declined at snowmelt [46, 47], and cold-adapted winter soil microbial communities died and were swiftly replaced by summer ones [48, 49]. Since we did not observe a sudden collapse of the microbial biomass at snowmelt [24], we consider that under a previous winter snow cover with stable soil temperatures >0 °C (Fig. S1), a specific winter-adapted microbial community does not develop nor dies at thaw. The dynamics and composition of the alpine microbiome is thus dependent on climatic conditions. Current global warming is already reducing the alpine winter snow cover [51], resulting in colder, often frozen soils. This may challenge the stability we observed and result in spring shifts of the microbial communities and their functions [52].

### Taxonomic and functional diversity

Our study challenges two commonly held assumptions concerning the diversity and the functional groups of the soil microbiome. The biases induced by “universal eukaryotic primers” during PCR overestimate the “SAR” (or Harosa) clade, particularly the ciliates, while strongly underestimating Amoebozoa (especially Conosa and the slime moulds), as previously signalled [53, 54]. RNA-based studies unequivocally agree on revealing Amoebozoa as an important (when not dominant) protistan lineage, alongside Rhizaria, in soil and litter [55-57]. In our study, amoebozoans not only dominate the protistan assemblage, but also played a major role as consumers (Fig. 6)—it follows that neglecting them will result in an incomplete view of the soil food web. Our results indubitably shows that the bacterial predators (Myxococcota essentially) outnumber the protistan predators. They play an essential, and mostly unrecognized, role in shaping the microbial communities (Fig. 6), as already noticed [58].

The low proportion of protistan parasites (9%) is consistent with a previous study in the Swiss Alps, where their relative abundance decreased at the altitudes at which our survey was conducted [47]. Consumers were also the most abundant in temperate regions [59]. This is in contrast to tropical soils where parasites dominated [60, 61], probably related to the high abundance and diversity of insects [60].

### Methodological discussion

It increasingly appears that metatranscriptomics might become the preferred method for molecular monitoring of complex environmental microbiomes. Using mock communities, it has been shown that rRNA-based methods outperform metagenomics in identifying taxa [62]. DNA-based surveys are inappropriate for monitoring short- to middle-term shifts, since they include a fair portion of dead organisms [63]. Possible biases are discussed in Note S1.

### Concluding remarks

The soil microbial food web in alpine environments shows striking seasonal dynamics, with biotic interactions explaining a higher proportion in variability of consumer turnover than soil properties (e.g. pH and carbon content) or topography (elevation, spatial difference between mountains). Our study stands out by applying metatranscriptomics to a large-scale ecological appraisal of the entire soil microbiota. We achieved a sequencing depth that surpasses the descriptive limits of classical amplicon-based approaches enabling inter-domain and inter-sample comparisons. We complement our protocol with trait-based approaches to enhance basic knowledge of the soil food web functioning. Thus, our study contributes to the understanding of the alpine ecosystem by showing the importance of the biotic interactions in shaping the seasonal dynamics of the soil microbiome.

## Supplementary Material

SupplementaryNote1_ycae028

TableS1DatabaseEnvironmenta_ycae028l

TableS2DatabaseTaxaTraits_ycae028

TableS3TresholdSummary_ycae028

TableS4SequencesObtained_ycae028

TableS5SummaryRDA_ycae028

TableS6Network_ycae028

FigS1GraphSummaryTemp_ycae028

FigS2PcoA_ycae028

FigS3RarefactionCurvesSummary_ycae028

FigS4AccumulationCurvesSummary_ycae028

FigS5BarChartSnowCoverage_ycae028

FigS6NetworksEdibleOnly_ycae028

## Data Availability

Sequencing data and raw sequences are available under NCBI BioProject PRJNA850398.
