# Supplementary material for: Biotic interactions explain seasonal dynamics of the alpine soil microbiome
Source: ISME Commun. 2024 Feb 28;4(1):ycae028. doi: 10.1093/ismeco/ycae028 (PMC10945362; doi:10.1093/ismeco/ycae028)
Supplement: SupplementaryNote1_ycae028 [file supplementarynote1_ycae028.pdf]

**Fiore-Donno et al. "Biotic interactions explain seasonal dynamics of the alpine soil microbiome".**

**Supplementary Note 1.** Additional information on taxonomic diversity and methodological discussion.

## Taxonomic diversity

Prokaryotes totalized 98.02% of the genera, with Bacteria and Archaea accounting for 99.94% and 0.06% of their sequences, respectively (Supplementary Table 2). Bacteria were dominated by three phyla, Acidobacteriota (26%), Proteobacteria (23%), and Actinobacteriota (16%). The Proteobacteria were mostly Gammaproteobacteria (~55%) and Alphaproteobacteria (~45%). The predatory Myxococcota represented 5.72% (Fig. 2A). In Archaea the phylum Crenarchaeota (95%) and the class Nitrososphaeria (94%) dominated (Supplementary Table 2).

Among eukaryotes, Fungi and Metazoa accounted for 36% of the sequences each, protists for 17%, and multicellular plants for 11% (Supplementary Table 2). More than one third of the protistan sequences were assigned to the phylum Amoebozoa (35%) (Fig. 2B). Among it, most of the sequences were identified as Conosa (19%), then Discosea (11%, mostly represented here by *Acanthamoeba*) and Tubulinea (5%). Conosa mainly was composed of Variosea and the slime-moulds Myxogastria (83% and 16% of the conosan sequences, respectively). In Tubulinea, the amoebae with shell (Arcellinida) and Euamoebida (mainly the genus *Copromyxa*, a dung-inhabiting amoeba) accounted for 37% and 25% of the tubulinean sequences, respectively. Rhizaria (23%) was in majority composed of Cercozoa (20%), and only of 2% of the mostly parasitic Endomyxa. Alveolata accounted for 21%, with 14% of Ciliophora and 6% of Apicomplexa. The Stramenopiles (also named Heterokonta, 7%), with 3% of microscopic brown algae (Ochrophyta), 2% of Sagenista (labyrinthulids) and 2% of Opalozoa (mostly parasitic, including the Oomycetes).

Among the animals (Metazoa), insects (41%) and ringed worms (34%) were most abundant (Fig. 2C), followed by nematodes (16%). Arthropoda, composed mostly of insects (41%) and Chelicerata (4%), among them mostly arachnids, accounted for 45% of the sequences. The microscopic rotiferans and tardigrads accounted for 2% each.

The fungal diversity was definitely dominated by Ascomycota (46%) (Fig. 2D), of which mostly Pezizomycotina (37%), which also makes up most of Ascomycota in terms of described species. The Mucoromycota (19%) was composed in majority of undetermined taxa and the genus *Morteriella*, a soil saprotroph. Basidiomycota (19%) was dominated by the Agaricomycotina (18%) and only 1% of the plant parasites Pucciniomycotina. The mostly arbuscular mycorrhizal Glomeromycota accounted for 6%, and the mostly saprophytic chytrids for 3%. Interestingly, one of the most abundant genera of fungi (6.6%) belonged to the recently described class Archaeorhizomycetes (*incertae sedis* in Ascomycota), a very elusive group that doesn't form fruiting bodies. Archaeorhizomyces were widespread and abundant in the Alps, since it was the most abundant genus (6.6% of fungal sequences), as in Pinto-Figueroa et al. (2019), where it represented 13.3.% of all fungal ITS sequences.

## Methodological discussion

### a. rRNA biases, copy numbers and microbial abundance

In our analyses, we broadly assume that the rRNA copy numbers, binned to the genus level, represent the abundance of these genera, which is tentative. There are indeed recognized biases, the first being sequencing errors overestimating the diversity, which we mitigated by deleting genera represented by one to three reads, and conducted, when necessary, analyses only on the most abundant taxa. Secondly, an undetermined proportion of wrong assignments is due to the inaccuracy of the reference databases, still containing mislabelled sequences. Since the higher the taxonomic level, the less likely are those errors, we conducted our analyses at the orders, classes and phyla levels. Thirdly, the amplification step during library preparation may be less efficient for

extremely A-T or G-C rich regions (Shi et al. 2021), which we could not test or evaluate in our study.

It is often assumed that metatranscriptomics only target active soil microbes, e.g. (Geisen et al. 2015; Harkes et al. 2019). While this is certainly true for messenger RNAs, it has been shown that dormant cells can also contain high numbers of ribosomes ((Blazewicz et al. 2013 and references therein). Therefore, rRNA-based studies of the soil microbiome, which is likely to include a high proportion of dormant cells, cannot provide information on microbial activity.

It is still currently unclear how to relate rRNA transcript numbers with microbial cell numbers for inferring abundances (Geisen et al. 2015; Petters et al. 2021; Söllinger et al. 2022). Biases are probably introduced by varying ribosomal RNAs copy numbers per cell, between prokaryotes and eukaryotes, between taxa and even inside taxa (Harkes et al. 2019). Recently, a down-regulation of the bacterial protein biosynthesis machinery has been observed in response to warming (Söllinger et al. 2022). At present, these biases are unquantifiable, but some hints suggest that they may only be cavils. A study showed that the cell abundance of small planktonic eukaryotes was correlated with the rDNA abundance (Gong et al. 2020) but for two species of ciliates, it varied with cell-size, so that the rRNA concentration was higher in smaller than larger cells (Fu et al. 2017). Accordingly, the ratio of the phospholipids' fatty acid-derived biomass of fungi to bacteria is 8.6 (6.7~11.0) in cold climates (He et al. 2020), while in metatranscriptomics bacteria dominated by large the counts of SSUs (Urich et al. 2008) and the present study. Thus, the higher biomass of soil fungi, which have larger cells than bacteria, is not mirrored by rRNA-based approach, and the results from both methods cannot be compared. General quantification methods are still wanting and will represent a huge step forward when available.

## b. Network analyses

In addition to the analyses conducted as described in the main text and showed in Fig. 6 - associations between consumers and consumers and preys in spring and summer (and Fig. S6, associations between preys only), we also tested the effect of selected environmental parameters on these networks, i.e. mountain, altitude, soil temperature, days under snow, soil water content, pH, microbial C, microbial N, microbial CN, Organic C, Dissolved N. The biotic associations of the obtained networks with and without environmental data were quite similar. Thus, FlashWeave's predictions appear to be robust against shared-niche biases, i.e. inflation of associations between taxa driven by environmental factors, even with missing environmental data.

**Co-occurrence networks of abundant phyla of consumers and preys, with selected environmental factors**. **a** Spring, under the snow. **b** Summer. The size of the nodes (dots) is proportional to the number of reads. Edges (connecting lines) represent positive (light grey) or negative (dark grey) correlations, with line width proportional to the number of correlations. Self-loops, taxa with a single edge and connections between preys are not shown. Note the similarity with networks shown in Fig. 6.

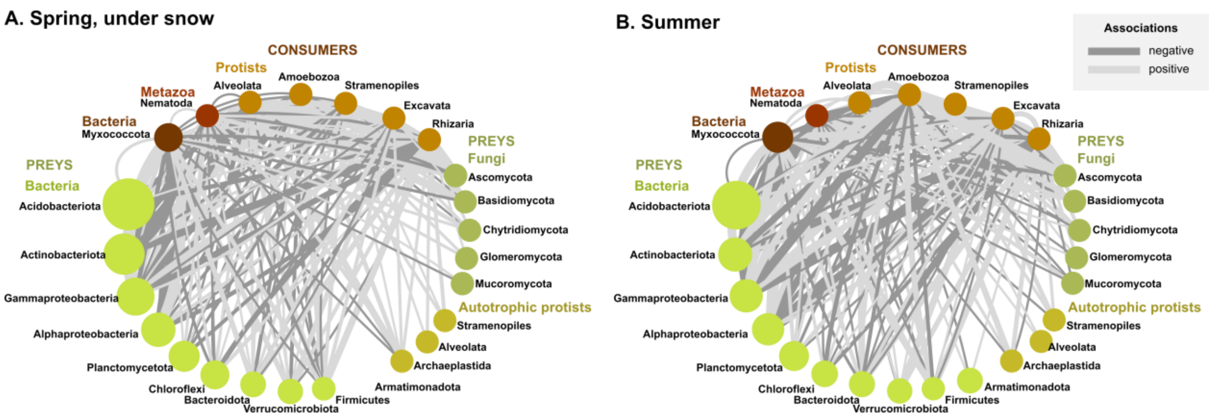

## Network metrics

| | Snow | Summer |
|---|---|---|
| **Number of Nodes** | 1869 | 2017 |
| **Number of Edges** | 2779 | 3003 |
| **Avg. Number of neighbors** | 2.998 | 3010 |
| **Network diameter** | 19 | 22 |
| **Network radius** | 12 | 12 |
| **Characteristic path length** | 7.723 | 7.694 |
| **Clustering coefficient** | 0.002 | 0.002 |
| **Network density** | 0.002 | 0.002 |
| **Network heterogeneity** | 0.467 | 0.483 |
| **Network centralization** | 0.004 | 0.003 |
| **Connected components** | 12 | 16 |

## References


Blazewicz, S. J., R. L. Barnard, R. A. Daly & M. K. Firestone (2013). Evaluating rRNA as an indicator of microbial activity in environmental communities: limitations and uses. *ISME J.* 7: 2061-2068.

Fu, R. & J. Gong (2017). Single cell analysis linking ribosomal (r)DNA and rRNA copy numbers to cell size and growth rate provided insights into molecular protistan ecology. *J. Euk. Microbiol.* 64: 885–896.

Geisen, S., A. Tveit, I. M. Clark, A. Richter, M. M. Svenning, M. Bonkowski, et al. (2015). Metatranscriptomic census of active protists in soils. *ISME J.* 9: 2178–2190.

Gong, F., G. Li, Y. Wang, Q. Liu, F. Huang, K. Yin, et al. (2020). Spatial shifts in size structure, phylogenetic diversity, community composition and abundance of small eukaryotic plankton in a coastal upwelling area of the northern South China Sea. *J. Plankton Res.* 6: 650-667.

Harkes, P., A. K. A. Suleiman, S. J. J. van den Elsen, J. J. de Haan, M. Holterman, E. E. Kuramae, et al. (2019). Conventional and organic soil management as divergent drivers of resident and active fractions of major soil food web constituents. *Sci. Rep.* 9: 13521.

He, L., J. L. Mazza Rodrigues, N. A. Soudzilovskaia, M. Barceló, P. Axel Olsson, C. Song, et al. (2020). Global biogeography of fungal and bacterial biomass carbon in topsoil. *Soil Biol. Biochem.*

Petters, S., V. Groß, A. Söllinger, M. Pichler, A. Reinhard, M. M. Bengtsson, et al. (2021). The soil microbial food web revisited: Predatory myxobacteria as keystone taxa? *ISME J.* 15: 2665-2675.

Pinto-Figueroa, E. A., E. Seddon, E. Yashiro, A. Buri, H. Niculita-Hirzel, J. Roelof van der Meer, et al. (2019). Archaeorhizomycetes spatial distribution in soils along wide elevational and environmental gradients reveal co-abundance patterns with other fungal saprobes and potential weathering capacities. *Front. Microbiol.* 10: 656.

Shi, H., Y. Zhou, E. Jia, M. Pan, Y. Bai & Q. Ge (2021). Bias in RNA-seq library preparation: Current challenges and solutions. *BioMed Res. Int.* 2021: 6647597.

Söllinger, A., J. Séneca, M. Borg Dahl, L. L. Motleleng, J. Prommer, E. Verbruggen, et al. (2022). Down-regulation of the bacterial protein biosynthesis machinery in response to weeks, years, and decades of soil warming. *Sci. Adv.* 8: eabm3230.

Urich, T., A. Lanzén, J. Qi, D. H. Huson, C. Schleper & S. C. Schuster (2008). Simultaneous assessment of soil microbial community structure and function through analysis of the meta-transcriptome. *PLoS ONE* 3: e2527.
