# Supplementary material for: Biotic interactions explain seasonal dynamics of the alpine soil microbiome
Source: ISME Commun. 2024 Feb 28;4(1):ycae028. doi: 10.1093/ismeco/ycae028 (PMC10945362; doi:10.1093/ismeco/ycae028)
Supplement: FigS1GraphSummaryTemp_ycae028 [file figs1graphsummarytemp_ycae028.pdf]

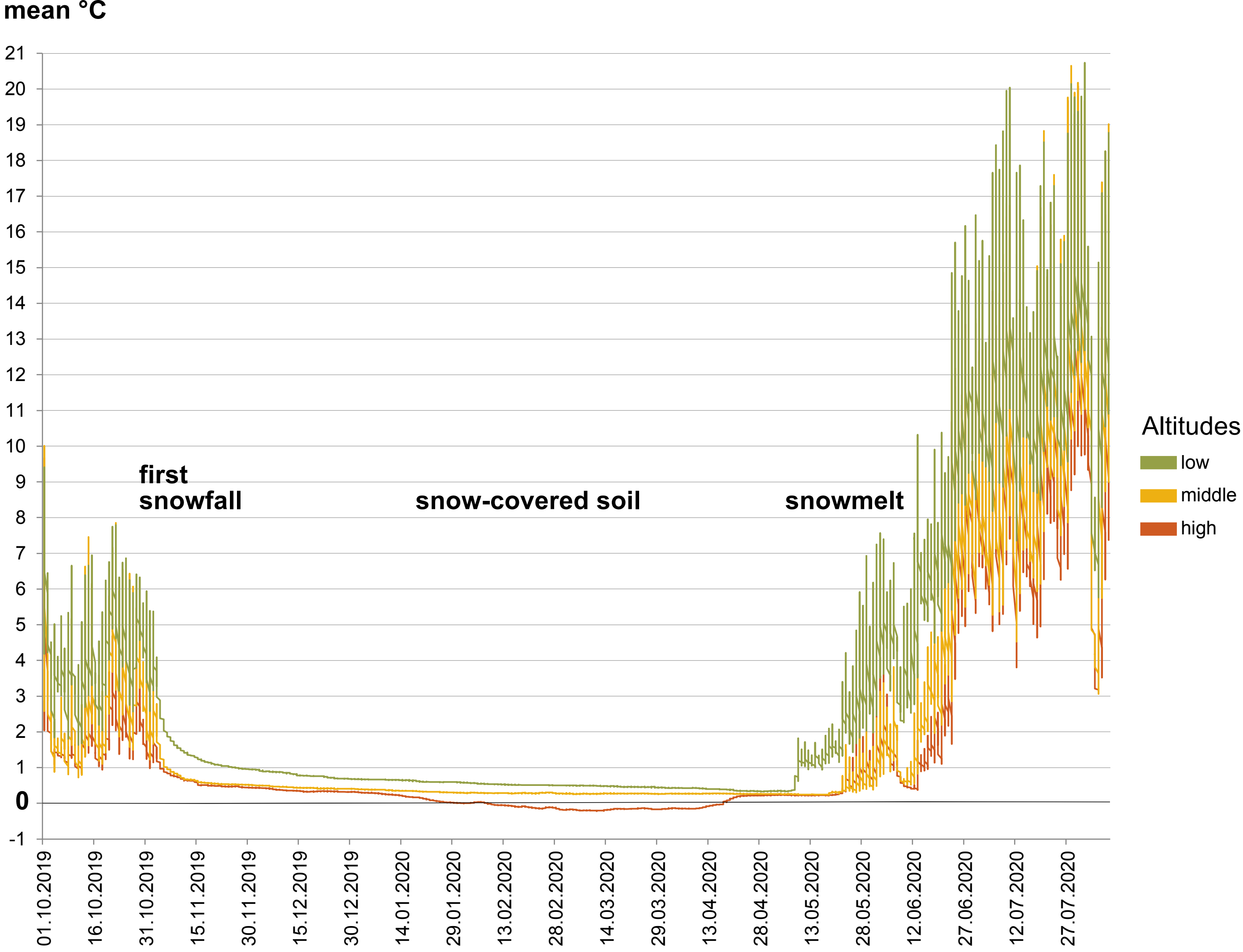

Figure S1. Soil temperature recorded every two hours from autumn 2019 to summer 2020, averaged by altitudinal categories. In winter the soil remained at a constant temperature just above zero degrees, except in the highest sites, where temperatures dropped slightly below 0°C but stayed above -1°C.
