# Supplementary material for: Biotic interactions explain seasonal dynamics of the alpine soil microbiome
Source: ISME Commun. 2024 Feb 28;4(1):ycae028. doi: 10.1093/ismeco/ycae028 (PMC10945362; doi:10.1093/ismeco/ycae028)
Supplement: FigS2PcoA_ycae028 [file figs2pcoa_ycae028.pdf]

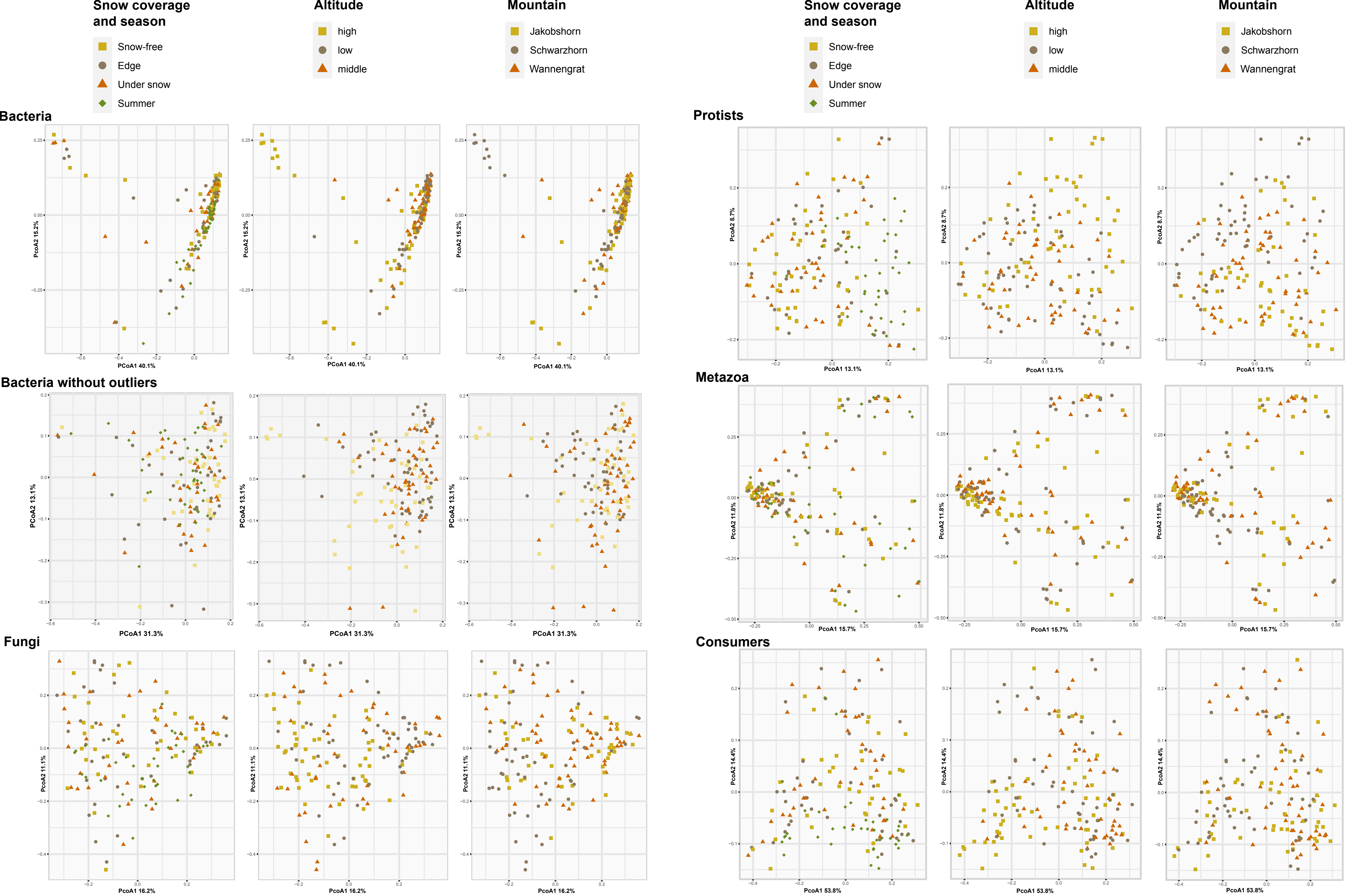

Figure S2. Principal Component Analysis of the Bray-Curtis dissimilarity indices of the main taxa and functions, showing that snow coverage, season, altitude and mountain have little influence in shaping the communities. The functional group "preys" was nearly identical to bacteria, and thus not shown.
