# Supplementary material for: Biotic interactions explain seasonal dynamics of the alpine soil microbiome
Source: ISME Commun. 2024 Feb 28;4(1):ycae028. doi: 10.1093/ismeco/ycae028 (PMC10945362; doi:10.1093/ismeco/ycae028)
Supplement: FigS3RarefactionCurvesSummary_ycae028 [file figs3rarefactioncurvessummary_ycae028.pdf]

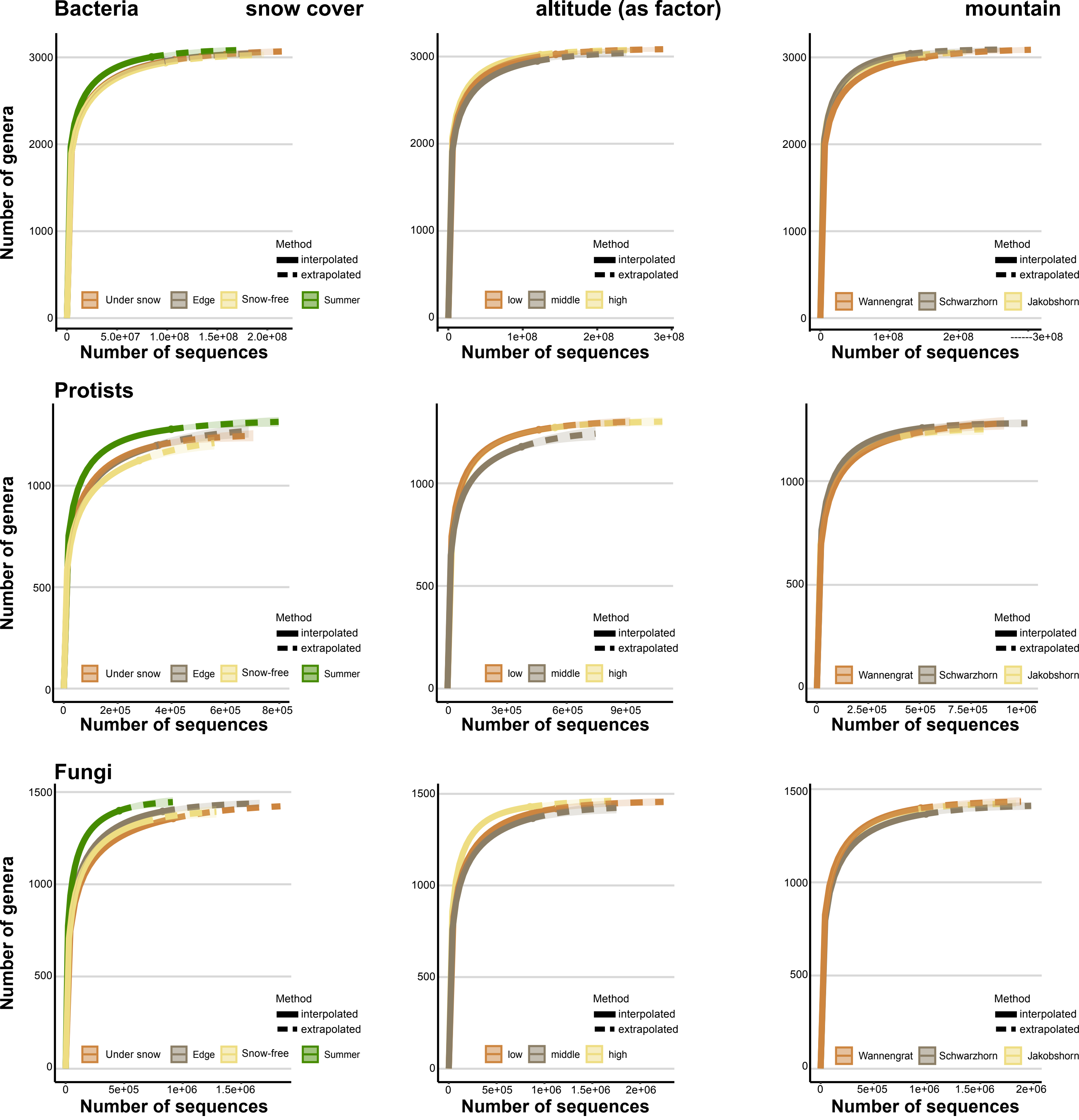

Figure S3. Rarefaction curves for bacteria, protists and fungi, by snow cover, altitude and mountain. They were calculated using the iNEXT package, on raw abundances, with a 97% confidence interval, 50 bootstraps and 50 knots.
