# Supplementary material for: Biotic interactions explain seasonal dynamics of the alpine soil microbiome
Source: ISME Commun. 2024 Feb 28;4(1):ycae028. doi: 10.1093/ismeco/ycae028 (PMC10945362; doi:10.1093/ismeco/ycae028)
Supplement: FigS5BarChartSnowCoverage_ycae028 [file figs5barchartsnowcoverage_ycae028.pdf]

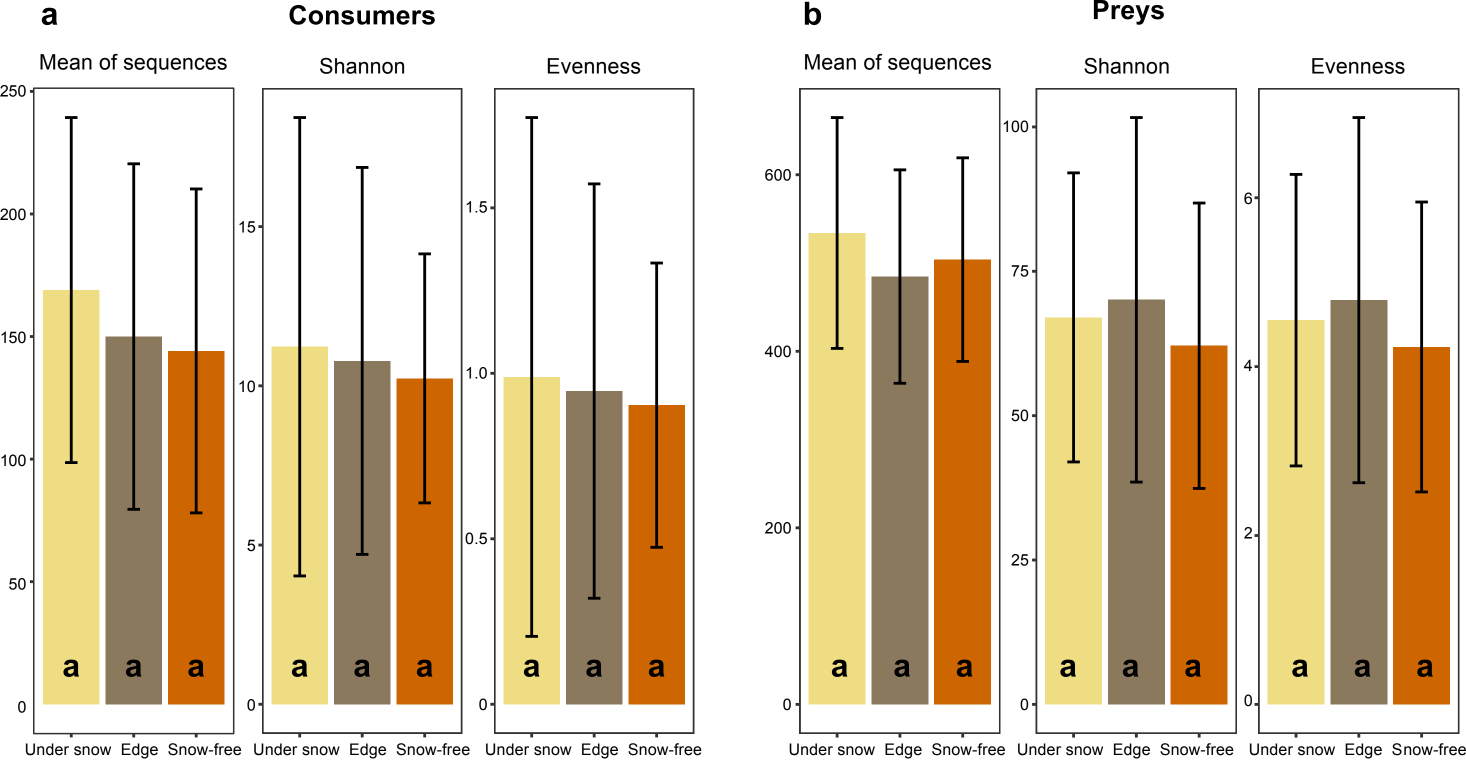

Figure S5. Variation in the mean of SSU sequences, diversity (Shannon index) and evenness, from the samples under the snow, at the edge of the snow patch and snow-free. There were no significant changes (Tukey-test, p-value⩽0.05), they are indicated by "a". Standard errors bars are shown. **a**, Consumers (predatory bacteria, heterotrophic and free-living protists, selected nematoda). **b**, Preys (non-predatory bacteria, fungi and autotrophic protists).
