# Supplementary material for: Biotic interactions explain seasonal dynamics of the alpine soil microbiome
Source: ISME Commun. 2024 Feb 28;4(1):ycae028. doi: 10.1093/ismeco/ycae028 (PMC10945362; doi:10.1093/ismeco/ycae028)
Supplement: FigS6NetworksEdibleOnly_ycae028 [file figs6networksedibleonly_ycae028.pdf]

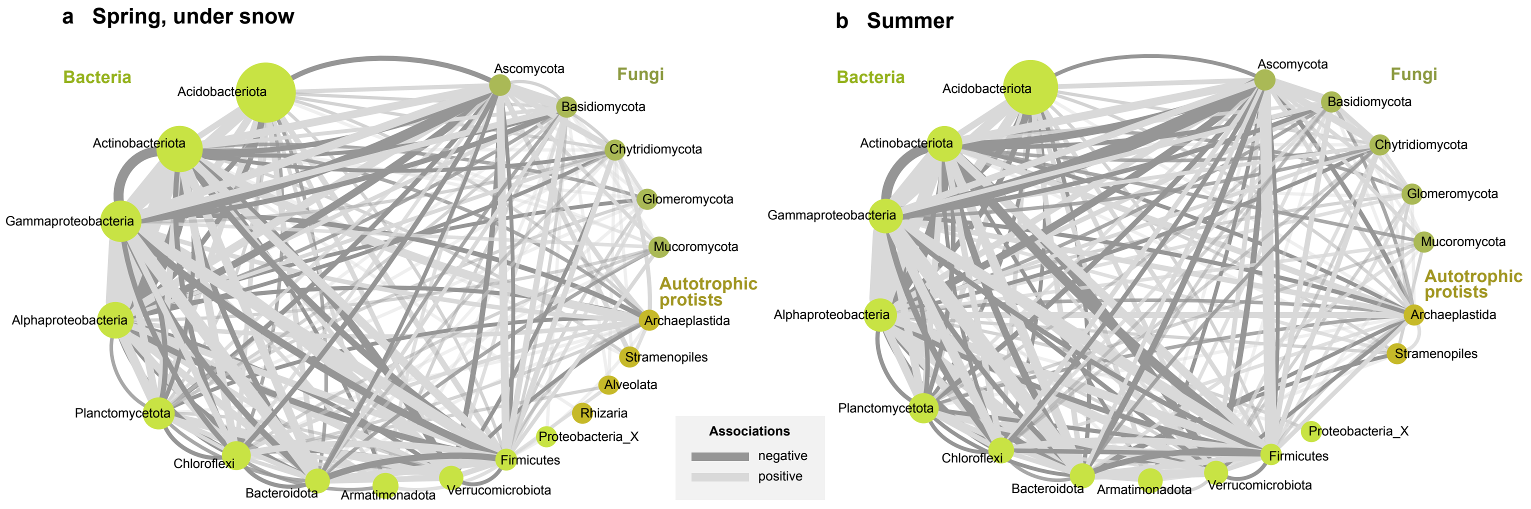

Figure S6. Co-occurrence networks of abundant phyla of preys. **a,** Spring, under the snow. **b**, Summer. The size of the nodes (dots) are proportional to the number reads. Edges (connecting lines) represent positive (light grey) or negative (dark grey) correlations, with line width proportional to the number of correlations. Self-loops and taxa with a single edge are not shown.
